# Effects by periodontitis on pristane-induced arthritis in rats

**DOI:** 10.1186/s12967-016-1067-6

**Published:** 2016-11-03

**Authors:** Kaja Eriksson, Erik Lönnblom, Gregory Tour, Anna Kats, Piotr Mydel, Pierre Georgsson, Catharina Hultgren, Nastya Kharlamova, Ulrika Norin, Jörgen Jönsson, Anna Lundmark, Annelie Hellvard, Karin Lundberg, Leif Jansson, Rikard Holmdahl, Tülay Yucel-Lindberg

**Affiliations:** 1 Division of Periodontology, Department of Dental Medicine, Karolinska Institutet, Box 4064, 141 04 Huddinge, Sweden; 2 Section for Medical Inflammation Research, Department of Medical Biochemistry and Biophysics, Karolinska Institutet, 171 77 Stockholm, Sweden; 3 Department of Laboratory Medicine, Karolinska Institutet at Karolinska University Hospital, Alfred Nobels Allé 8, 141 83 Huddinge, Sweden; 4 Broegelmann Research Laboratory, Department of Clinical Science, University of Bergen, The Laboratory Building, 5th Floor, 5021 Bergen, Norway; 5 Rheumatology Unit, Department of Medicine, Karolinska University Hospital, Solna, Rheumatology Clinic D2:01, 171 76 Stockholm, Sweden; 6 Malopolska Centre of Biotechnology, Jagiellonian University, Gronostajowa 7a, 30-387 Kraków, Poland; 7 Department of Periodontology at Eastmaninstitutet, Stockholm County Council, Dalagatan 11, 113 24 Stockholm, Sweden; 8 Center for Medical Immunopharmacology Research, Southern Medical University, Guangzhou, China

**Keywords:** Arginine gingipain, Citrullinated peptide, Cytokine, Inflammation, Peptidylarginine deiminase, Periodontitis, Pristane-induced arthritis, *Porphyromonas gingivalis*

## Abstract

**Background:**

An infection-immune association of periodontal disease with rheumatoid arthritis has been suggested. This study aimed to investigate the effect of pre-existing periodontitis on the development and the immune/inflammatory response of pristane-induced arthritis.

**Methods:**

We investigated the effect of periodontitis induced by ligature placement and *Porphyromonas gingivalis* (*P. gingivalis*) infection, in combination with *Fusobacterium nucleatum* to promote its colonization, on the development of pristane-induced arthritis (PIA) in rats (Dark Agouti). Disease progression and severity of periodontitis and arthritis was monitored using clinical assessment, micro-computed tomography (micro-CT)/intraoral radiographs, antibody response, the inflammatory markers such as α-1-acid glycoprotein (α-1-AGP) and c-reactive protein (CRP) as well as cytokine multiplex profiling at different time intervals after induction.

**Results:**

Experimentally induced periodontitis manifested clinically (P < 0.05) prior to pristane injection and progressed steadily until the end of experiments (15 weeks), as compared to the non-ligated arthritis group. Injection of pristane 8 weeks after periodontitis-induction led to severe arthritis in all rats demonstrating that the severity of arthritis was not affected by the pre-existence of periodontitis. Endpoint analysis showed that 89% of the periodontitis-affected animals were positive for antibodies against arginine gingipain B and furthermore, the plasma antibody levels to a citrullinated *P. gingivalis* peptidylarginine deiminase (PPAD) peptide (denoted CPP3) were significantly (P < 0.05) higher in periodontitis rats with PIA. Additionally, there was a trend towards increased pro-inflammatory and anti-inflammatory cytokine levels, and increased α-1-AGP levels in plasma from periodontitis-challenged PIA rats.

**Conclusions:**

Pre-existence of periodontitis induced antibodies against citrullinated peptide derived from PPAD in rats with PIA. However, there were no differences in the development or severity of PIA between periodontitis challenged and periodontitis free rats.

**Electronic supplementary material:**

The online version of this article (doi:10.1186/s12967-016-1067-6) contains supplementary material, which is available to authorized users.

## Background

Periodontitis is one of the most common infectious inflammatory diseases of mankind, affecting up to 30% of the world population [1, 2]. Epidemiological and clinical studies have linked this highly prevalent disease to other systemic inflammatory diseases such as cardiovascular diseases, chronic obstructive pulmonary disease, diabetes, obesity and rheumatoid arthritis (RA) [3–7]. In periodontitis, *P. gingivalis* has been demonstrated to be a key pathogen in the shift in composition of tooth-surface microbiota, from commensal to pathogenic [8]. In humans, infection with *P. gingivalis* and other important periodontal pathogens of the “red complex” [9, 10], in combination with additional microorganisms that promote their colonization such as *Fusobacterium nucleatum* (*F. nucleatum*) [11], initiates an inflammatory response in the periodontium, which may become chronic and lead to destruction of tooth-supporting structures. The signs of an on-going inflammation are visible not only locally in the periodontal pockets but also systemically [12] as elevated levels of inflammatory mediators are present in the blood of patients with periodontitis [13]. This may have a direct bearing on the development of atherosclerotic plaque and aggravation of other chronic inflammatory diseases [14], including RA, reported to be increased in population affected by periodontitis [15, 16].

A potential relationship between RA and periodontitis has been proposed since these two diseases, although different in terms of their etiological background, demonstrate several similarities including shared risk factors such as smoking and pathophysiological features including a similar set of effector inflammatory cells, pro-inflammatory cytokines, and other mediators that drive bone destruction [8, 17–21]. Up to 1% of the population worldwide suffers from RA, a painful chronic inflammatory autoimmune condition, where synovial joint inflammation destroys cartilage and bone causing irreversible joint damage [22]. The etiology of RA is not yet fully understood, however a complex interplay of genetic, environmental, hormonal and infectious risk factors are involved [22]. Moreover, antibodies to citrullinated protein antigens (ACPAs) have a high disease specificity and are associated with more aggressive disease course and joint destruction in RA [23]. Therefore, the processes linked to the generation of ACPAs are an area of interest in studies investigating the pathogenesis of this disease. Intriguingly, *P. gingivalis* secretes a unique bacterial enzyme, peptidylarginine deiminase (PAD), able to citrullinate proteins [17]. Citrullinated fibrin in synovium of RA patients is a major target for RA-specific ACPAs, making *P. gingivalis* PAD (denoted PPAD) a potential contributor to the arthritis-related immune response [17, 24, 25]. PPAD acts together with another major virulence factor, known as arginine gingipain B (RgpB), an arginine-specific extracellular protease expressed on the surface of the bacterial outer membrane [25]. RgpB has been shown to be essential for the ability of *P. gingivalis* to citrullinate peptides since the citrullination by PPAD, i.e. conversion of peptidylarginine into peptidylcitrulline, occurs after degradation by RgpB [25]. Moreover, antibody levels to RgpB are elevated in patients with RA compared to controls without RA [26]. According to an etiological hypothesis, actions of PPAD lead to a chronic exposure of citrullinated proteins in the inflamed periodontium, which triggers loss of immune tolerance and ACPA production [17]. Therefore *P. gingivalis* could represent a link between periodontitis and RA. In support of this hypothesis, it has been shown that citrullinated proteins are present in the inflamed periodontium and ACPAs have been detected in sera from patient with periodontitis [27, 28]. Moreover, it has been shown that ACPAs targeting human citrullinated α-enolase cross-react with *P*. *gingivalis* enolase, creating the basis of a molecular mimicry hypothesis between periodontitis and RA [18]. Also, antibodies against *P. gingivalis*, as well as *P. gingivalis* DNA, has been found in serum and synovial fluid in patients with RA [29, 30]. In 2010, Bartold et al. showed that induction of chronic inflammation by implanting polyurethane sponges impregnated with *P. gingivalis* resulted in rapid development of severe arthritis in Dark Agouti (DA) rats [31]. Additionally, rats with mycobacteria-induced adjuvant arthritis have demonstrated periodontal bone loss and elevated levels of matrix metalloproteinases (MMPs), tumor necrosis factor-α (TNF-α) and interleukin-1β (IL-1β) in gingival tissue [32]. Although several studies indicate that there is a relationship between periodontitis and RA, recent reviews conclude that a direct role of *P. gingivalis* in this temporal relationship still remains elusive [33]. The aim of this study was therefore to explore the effect of pre-existing experimental periodontitis, induced by ligatures and periodontal pathogens *P. gingivalis* in combination with *F. nucleatum* on the development of arthritis, induced by pristane, a well-established model for RA [34].

## Methods

### Animals

Twenty-six inbred adult male Dark Agouti (DA) rats (165–220 g) were included in this study, to investigate the effect of pre-existing periodontitis, induced by ligature and *P. gingivalis* infection in combination with *F. nucleatum* on the development of arthritis. The animals were bred and kept at the environmentally standardized animal facility, at Medical Inflammation Research, under specific pathogen free conditions, as previously described [35]. The rats were housed in individually ventilated cages (IVC rack), and received water and standard pellets ad libitum. Before starting the study, approval was obtained from the ethical committee (the Stockholm North Animal Ethics Committee, approval number Dnr N67/10 and N143/10). The rats were randomly divided into groups: PA—the experimental periodontitis group with induced arthritis (n = 10) and A—the arthritis induced group without periodontitis (non-ligated) (n = 12). Additionally, a group of healthy control rats without arthritis or periodontitis (non-ligated and non PIA-induced) (n = 4) was included. One rat from the PA group was excluded in the beginning of the experiment due to gender mix up (female). In the PA group induction of experimental periodontitis was performed for 8 weeks prior to pristane injection, using a ligature model in combination with recurring swabs with *P. gingivalis* and *F. nucleatum* (known to promote colonization of *P. gingivalis* adjacent to the affected teeth) [11] in order to facilitate a more generalized form of oral infection. After 8 weeks of experimental periodontitis, PIA was induced in both PA and A group and the development of arthritis was monitored for an additional 7 weeks. After 15 weeks, evaluated as experimental endpoint, all animals were euthanized using CO_2_.

### Experimental periodontitis

For induction of experimental periodontitis (PA group), the rats were anesthetized using isoflurane, and 4-0 silk ligatures (Johnson & Johnson International New Brunswick, NJ, USA) were placed around the cervical part of the second upper molars on both sides and knotted mesio-palatally [36]. Every 10 days, a dental examination was performed where the ligatures were controlled and tooth mobility was assessed by a trained dentist, and by two dentists at endpoint, to supervise the course of the experimental periodontitis. In case of partially loosened or missing ligatures, new ligatures were placed. To facilitate the development and progression of periodontitis, each dental examination was followed by swabs containing *P. gingivalis* CCUG 14449 in combination with *F. nucleatum* ATCC 10953 (known to promote the colonization of *P. gingivalis*) [11] reconstituted in 4% Gantrez copolymer medium (International Specialty Products, Köln, Germany) to promote bacterial adhesion and retention in the oral cavity. Additional swabs were administered once more in-between the times for dental examination. Rats in the arthritis non-ligated group (A) and the healthy control group received swabs with 4% Gantrez medium only.

The severity of experimentally induced periodontitis was monitored using a clinical manifestation grading scale of tooth mobility (modified from Lindhe and Nyman 1975) [37], as well as by assessment of radiological alveolar bone levels at endpoint (week 15) [36]. At all consecutive time points the tooth mobility, of the left and right second maxillary molars, was assessed in all animals. Tooth mobility grading (with a maximum score of 6 points/rat) represented 1, 2 or 3 points corresponding to mobility grade 1 = slight mobility in buccolingual direction, 2 = mobility in buccolingual and mesiodistal directions or 3 = vertical mobility [38]. Zero (0) points indicated no clinical manifestation of periodontitis. The total mean score (±SE) for each group was calculated for each time point. For assessment of radiological alveolar bone levels, the upper jaws of the experimental animals were separated at the intermaxillary suture, X-rayed using a standard intraoral X-ray unit (Planmeca, Helsinki, Finland) and analyzed using the Planmeca Romexis software, as previously described [36]. The separated jaws were placed on a Planmeca ProSensor and exposed for 0.16 s to 60 kV and 8 mA. The alveolar bone levels were measured from a straight line marking of the occlusal surface of the upper second molars, to the marginal bone at the mesial and distal interproximal sites [36]. The measurements were performed blindly and independently by two calibrated dentists. For further illustration of the alveolar bone loss and corresponding arthritic changes in limbs, the intact sculls of representative animals were scanned together with front and hind paws using micro-computed tomography (micro-CT).

### Experimental arthritis

To induce PIA, we gave a single intradermal injection at the dorsal side of tail with 100 µl of pristane (2,6,10,14-tetramethylpentadecane, 95% Arcos Organics, Morris Plains, NJ, USA) 8 weeks after experiment start in both the PA group and non-ligated A group. Assessment of arthritic changes in limbs was performed in the regions of interest to monitor the destruction of articular cartilage and ankylosis of the joints, as well as inflammatory response of the synovium. A visual scoring system of 1–60 points per rat was used as described previously [39]. In brief, 1 point was given for each knuckle or toe with inflammation and up to 5 points for an affected ankle or wrist, in total 15 points per paw. All scoring was performed blindly and independently by two examiners. Weight changes of each rat were monitored during the course of the experiment as an objective measure of disease severity [35].

### Micro-computed tomography

Fixed crania, front and hind paws were scanned using nanoScan PET/CT (Mediso Ltd, Budapest, Hungary) to illustrate the alveolar bone loss and arthritic changes in limbs. The three-dimensional images of the limbs and maxilla were reconstructed and calibrated to the Hounsfield scale with an effective isotropic voxel size of 0.02 mm. Imaging of paws was conducted with Able-Software 3D-Doctor.

### Bacterial culture and preparation

*Porphyromonas gingivalis* (CCUG 14449) and *F. nucleatum* (ATCC 10953) obtained from Culture Collection, University of Gothenburg, Sweden and American Type Culture Collection, Germany, respectively, were cultured at 37 °C, on anaerobic blood agar plates (Brucella/Brucella supplemented with heme and vitamin K). Bacterial swab mixtures of *P. gingivalis* and *F. nucleatum* were prepared at the density of 2 × 10^8^ and 1 × 10^8^ cells/ml (counted using Bürker counting chamber), respectively. The bacterial mixtures (transported to the animal facility in an anaerobic cultivation system) were vortexed prior application to achieve homogenous distribution and applied around the second upper molar, 50 µl bacterial mixture on each side (100 µl/rat). In parallel, additional bacterial mixtures were also prepared and transported in an anaerobic cultivation system to detect the survival of *P. gingivalis*/*F. nucleatum*. For the detection of bacteria at endpoint, ligature and mucosal samples were cultured. Mucosal swabs were plated on Brucella agar for *P. gingivalis* culture, and on Brucella agar supplemented with heme and vitamin K for *F. nucleatum.* Ligature samples transported in 1 ml FAB were resuspended in PBS (dilution 1:100), and 100 µl of each sample was then plated for *P. gingivalis*/*F. nucleatum* on corresponding agar plates. Each ligature sample was also cultured undiluted. Cultures were incubated at 37 °C in anaerobic conditions for 5–7 days. Colonies of potential *P. gingivalis*/*F. nucleatum* were regrown on new agar plates, for purification and identification. Gram staining was used to confirm identification. Brucella agar (MIK1533) and Brucella agar supplemented with heme and vitamin K (MIK3657) were purchased from Substrate unit, Karolinska University Hospital (Huddinge, Sweden).

### Sampling and processing

Blood samples (0.2 ml) were collected from the tail of all the rats at the start and during consecutive controls (every 10 days) of the experimental period of 15 weeks. In addition, blood from the heart was collected by cardiac puncture (10 ml) at the end of the experimental period. Ligatures, both loosened during the course of experiment and those collected at the end of experiment were transferred either into fastidious anaerobe broth (FAB) and transported to the laboratory on ice (right side) or snap frozen in liquid nitrogen and transferred to −80 °C for subsequent analysis (left side). At the end of experiment, week 15, mucosal surfaces (buccal and dorsum of the tongue) were swabbed for bacteria using a sterile cotton swab. Swabs were placed into FAB, transported on ice and stored at 4 °C until analysis (within 24 h). Both hind paws, right front paw and heads following decapitation and skin removal, were all collected in 4% phosphate buffered formaldehyde and stored at 4 °C until micro-CT. Left front paw was snap frozen in liquid nitrogen and transferred to −80 °C for real-time qPCR analysis.

### Extraction of DNA from joints and dental ligatures

Genomic DNA (gDNA) was isolated and purified from the silk ligatures or tissues using QIAamp DNA Mini Kit (Qiagen, USA) [40]. Briefly, ligatures and bacterial cells were pelleted by centrifugation, resuspended in tissue lysis buffer (ATL) followed by overnight lysis with proteinase K (Qiagen) at 56 °C and purified using ethanol-containing buffers according to manufacturer’s instructions. gDNA was eluted in nuclease-free water, followed by spectrophotometric quantification and quality assessment (A_260_) with NanoVue spectrophotometer (GE Healthcare Bio-Science, Uppsala, Sweden) and stored at −20 °C until amplification by real-time qPCR. For gDNA isolation from inflammatory infiltrates, metacarpophalangeal joints including connective tissue of the joint capsule were dissected and homogenized by gently crushing after submerging in liquid nitrogen followed by proteinase K treatment as above. Purified diluted gDNA from the *P. gingivalis* strain used in this animal study, CCUG 14449, as well as strains 33277 and W83 were used as positive controls.

### Detection of *P. gingivalis* with real-time qPCR analysis

PCR reactions were performed on DNA samples from ligatures and tissues using the universal primer 16S to detect the presence of non-specific bacterial DNA species and with *P. gingivalis*-specific primer to detect the presence of *P. gingivalis* [41]. The assays were carried out using 7500 Fast Real-Time qPCR System (Applied Biosystems, Foster City, CA USA). Triplicate samples were assayed in a total volume of 20 μl, containing 40 ng of template gDNA solution, TaqMan Universal PCR Master Mix (2x) (Applied Biosystems, Foster City, CA, USA), and the specific set of primers (final concentration 18 μM) and probe (final concentration 5 μM) (Cybergene AB, Solna, Sweden), corresponding to 900 nM of forward and reverse primer and 250 nM of the probe at the final 1x concentration. After an initial incubation step of 2 min at 50 °C and denaturation for 10 min at 95 °C, 40 PCR cycles (95 °C for 15 s, 60 °C for 1 min) were performed. Three biological replicates were used for calculating the means and standard deviations for each ligature/animal (total n = 9). The experiment was repeated on two different occasions, on loosened ligatures and those collected at the end of experimental protocol. The primers and probes used for the analysis were: *P. gingivalis* forward primer 5′-GCG CTC AAC GTT CAG CC-3′, reverse primer 5′-CAC GAA TTC CGC CTG C-3′, probe 5′-6FAM-CAC TGA ACT CAA GCC CGG CAG TTT CAA-TAMRA-3′; 16S DNA universal forward primer 5′-TGG AGC ATG TGG TTT AAT TCG A-3′, reverse primer 5′-TGC GGG ACT TAA CCC AAC A-3′, probe 5′-6FAM-CAC GAG CTG ACG ACA RCC ATG CA-TAMRA-3′.

### Measurements of α-1-acid glycoprotein (α-1-AGP), C-reactive protein (CRP), arginine gingipain B (RgpB), α-enolase (CEP-1) and citrullinated PPAD peptide (CPP3) levels

Plasma levels of α-1-AGP and CRP were assessed using ELISA kits according to the manufacturer protocols (Life Diagnostics Inc. West Chester, PA, USA and Sigma-Aldrich, St.Louis, MO, USA, respectively). Briefly, plasma rat samples were diluted 1:40,000 and incubated in microtiter wells with solid phase affinity purified anti-rat α-1-AGP antibodies for 45 min or biotinylated rat CRP antibodies for 1 h, followed by incubation with horseradish peroxidase conjugate and subsequent detection with tetramethylbenzidine (TMB) reagent. The optical densities of α-1-AGP and CRP were measured spectrophotometrically and plasma levels were calculated using mean absorbance from standard curve and reference standards at 450 nm. The concentrations of α-1-AGP and CRP were expressed as mean (μg/ml ± SE).

Citrullinated and uncitrullinated arginine peptides (synthetic) from α-enolase (CEP-1/REP-1) and PPAD (CPP3/RPP3), as well as antibodies against RgpB protein were analyzed using an in-house peptide ELISA [42]. Briefly, plasma samples were diluted 1:2 for CPP3/RPP3, 1:20 for CEP-1/REP-1 and 1:50 for RgpB in buffer (1% BSA, 350 mM NaCl, 10 mM Tris–HCl, 1% Triton X-100, 0.1% SDS) and incubated overnight in high-binding plates (MaxiSorp, Nunc) coated with CPP3/RPP3 (10 μg/ml), CEP-1/REP-1 (2.5 μg/ml) or RgpB protein (3.4 μg/ml). Plates were washed in PBS-Tween (0.05%) and incubated with HRP-conjugated goat anti-rat IgG (Jackson Immuno Research Inc, West Grove, PA, USA) at room temperature for 1 h. Antibodies, measured in arbitrary units (expressed as mean AU/ml ± SE), were detected using TMB reagent (Sigma-Aldrich, St. Louis, MO, USA) and calculated based on a standard curve from a serially diluted highly positive serum pool. Absorbance was measured at 450 nm.

### Cytokine profiling

The endpoint plasma concentrations of the cytokines [sensitivities in square brackets] tumor necrosis factor-α (TNF-α) [3 pg/ml], interleukin(IL)-1α [1 pg/ml], IL-1β [2 pg/ml], IL-2 [3 pg/ml], IL-4 [1 pg/ml], IL-5 [6 pg/ml], IL-7 [0.4 pg/ml], IL-10 [5 pg/ml], IL-12p70 [0.7 pg/ml], IL-13 [0.9 pg/ml], IL-17A [0.1 pg/ml], IL-18 [4 pg/ml], macrophage colony-stimulating factor (M-CSF) [0.4 pg/ml], monocyte chemotactic protein 1 (MCP-1) [4 pg/ml], macrophage inflammatory protein (MIP)-3α [0.7 pg/ml], regulated on activation normal T cell expressed and secreted (RANTES) [3 pg/ml], erythropoietin (EPO) [8 pg/ml], granulocyte–macrophage colony-stimulating factor (GM-CSF) [0.6 pg/ml], growth-related oncogene/keratinocyte-derived chemokine (GRO/KC) [0.6 pg/ml] and vascular endothelial growth factor (VEGF) [0.3 pg/ml] were analyzed using multiplex Bio-Plex Pro™ Rat Cytokine 24-plex Assay and Bio-Plex 200 system (Bio-Rad, Sweden). The concentrations of the cytokines were expressed as mean (range) pg/ml.

### Statistical analysis

The statistical analyses were performed by using the statistical package IBM SPSS Statistics 21.0. For the alveolar bone measurements, the results are expressed as mean ± SE and values calculated using the results of measurements from two calibrated dentists. Student’s *t* test (two-tailed) was used to analyze differences in alveolar bone level between the groups. Comparisons between treatment groups according to separate cytokine levels, α-1-AGP and CRP levels in blood plasma and differences for citrullinated/uncitrullinated peptide levels in plasma were performed by using Wilcoxon rank sum test. P < 0.05 was considered statistically significant.

## Results

### Ligature and periodontal pathogen-induced periodontitis

**Fig. 1:**
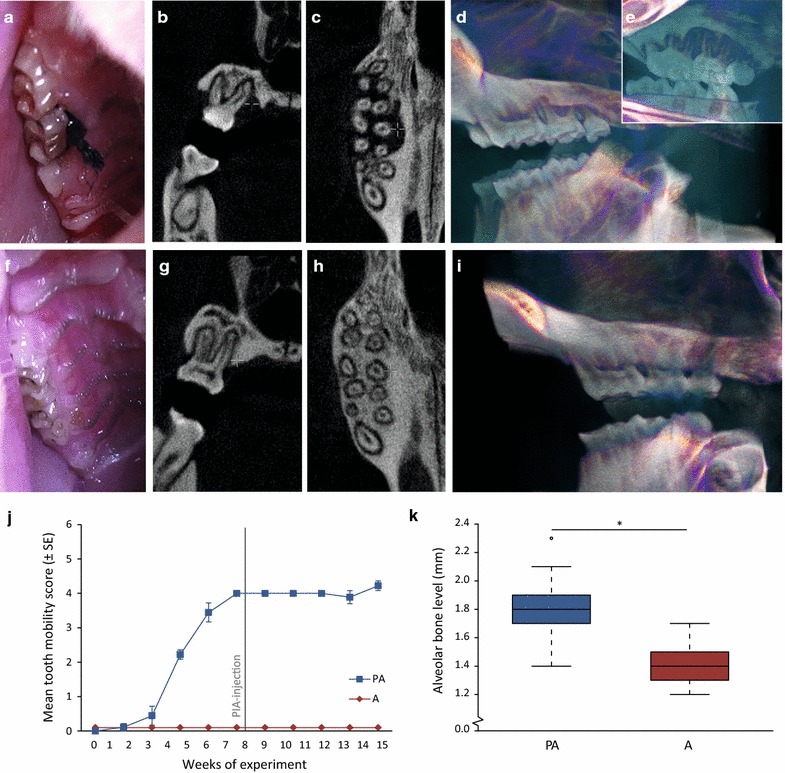
Clinical manifestation of periodontitis and radiographic assessment of bone loss. **a** Intraoral photograph of the ligated second upper molar in a rat with arthritis and experimental periodontitis. **b** Radiograph image of bone volume around a second upper molar, **c** micro-CT data (horizontal section) indicating bone loss around the upper molars and** d**,** e** 3D rendering of micro-CT data of bone volume around maxillary molars in an arthritis rat with experimental periodontitis. **f** Intraoral photograph of the non-ligated second upper molar of an arthritis rat without induced periodontitis. **g** Radiograph image of bone volume around the second upper molar, **h** micro-CT data (horizontal section) indicating bone loss around the upper molars and **i** 3D rendering of micro-CT data of bone volume around maxillary molars in a rat without induced periodontitis. **j** Mean tooth mobility score (±SE) in arthritis rats with periodontitis (n = 9) and without periodontitis (n = 12) throughout the experimental period of 15 weeks. **k** Distribution of mean alveolar bone level at endpoint in arthritis rats with periodontitis (n = 8) and without periodontitis (n = 10). ^*^P < 0.05 was considered statistically significant. *Circle* indicates outliers. *Gray line* indicates time point of pristane injection. *PIA* pristane-induced arthritis, *PA* rats with experimental periodontitis and PIA, *A* rats with PIA without periodontitis

The intraoral and radiograph dental images of DA rats with PIA and experimental periodontitis (PA group), and PIA without periodontitis (A group) are illustrated in Fig. 1a–i. The evaluation of ligature and periodontal pathogen-induced periodontitis, showed significant tooth mobility and alveolar bone loss after 15 weeks of experimental period, endpoint (Fig. 1b–e, j, k). Severe localized periodontitis, as assessed by tooth mobility score, manifested in all periodontitis-induced animals by week 6, prior to injection of pristane, and progressed further reaching plateau at week 9 (Fig. 1j). In contrast, neither the arthritis group without induced periodontitis (non-ligated) (A) nor the healthy controls rats (no periodontitis and no arthritis) showed any clinical signs of periodontitis, in terms of tooth mobility, during the course of the experiment (Fig. 1j and data not shown, respectively). Micro-CT scanning and assessment of alveolar bone level in intraoral radiographs showed significant (P < 0.05) loss of alveolar bone at the end of experiment for the PA group (mean alveolar bone level 1.8 ± 0.02 mm), as compared to the non-ligated arthritis group (mean alveolar bone level 1.4 ± 0.02 mm) (Fig. 1b–e, g–i, k). The alveolar bone level of the healthy control group (no periodontitis and no arthritis) was 1.5 ± 0.03 mm demonstrating no significant differences between the arthritis and healthy control group at the end of experiment.

To confirm presence of *P. gingivalis* and *F. nucleatum* in the site of inflammation, oral swabs and ligatures were collected at the end of the experiment (week 15) and cultured on respective plates in anaerobic conditions. Mixed bacterial flora was present in all of the samples, generating around 5 × 10^2^ CFU per sample (data not shown). *F. nucleatum* was identified in 6 out of 9 challenged animals (2 in oral swabs, 4 in ligature cultures). No viable *P. gingivalis* were detected in the cultures at endpoint although at the time of application of the bacterial swabs live *P. gingivalis* were detected using cultivation method (data not shown). DNA samples isolated from ligatures at endpoint were tested using *P. gingivalis*-specific real-time qPCR primers. Using that technique, we were able to confirm the presence of *P. gingivalis* DNA in ligatures from all 9 challenged animals (Additional file 1: Figure S1a, b). Due to the fact that the presence of *P. gingivalis* DNA in synovial tissue of patients with active periodontitis have previously been reported [43], we also tested DNA isolated from metacarpophalangeal joint tissue of challenged animals. However, we were not able to detect any traces of *P. gingivalis* in these samples (data not shown).

### Effect of pre-existing periodontitis on the development of pristane-induced arthritis

**Fig. 2:**
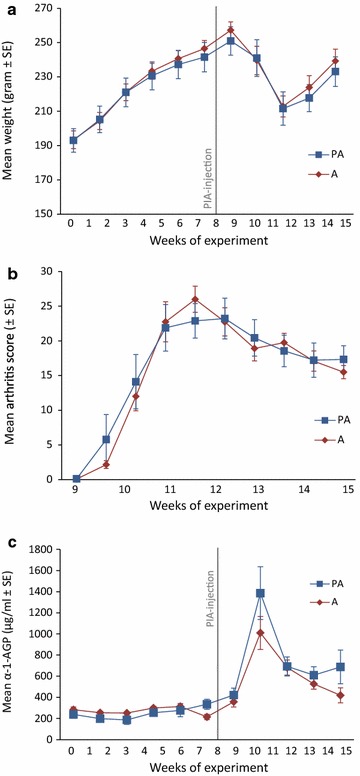
Effect of pre-existing periodontitis on weight changes, arthritis disease severity and α-1-AGP levels. **a** Mean changes in weight (grams ± SE) in arthritis rats with and without periodontitis throughout the experimental period of 15 weeks. **b** Mean arthritis disease severity score (±SE) of front and hind paws in arthritis rats with and without periodontitis, up to 7 weeks after pristane injection. **c** Mean plasma concentrations (μg/ml ± SE) of α-1-AGP in arthritis rats with and without periodontitis throughout the experimental period of 15 weeks. The results are presented as mean values (±SE) for the groups PA (n = 9) and A (n = 12) at different time-points throughout the experiment. *Gray line* indicates time point of pristane injection. *PIA* pristane-induced arthritis, *PA* rats with experimental periodontitis and PIA, *A* rats with PIA without periodontitis

**Fig. 3:**
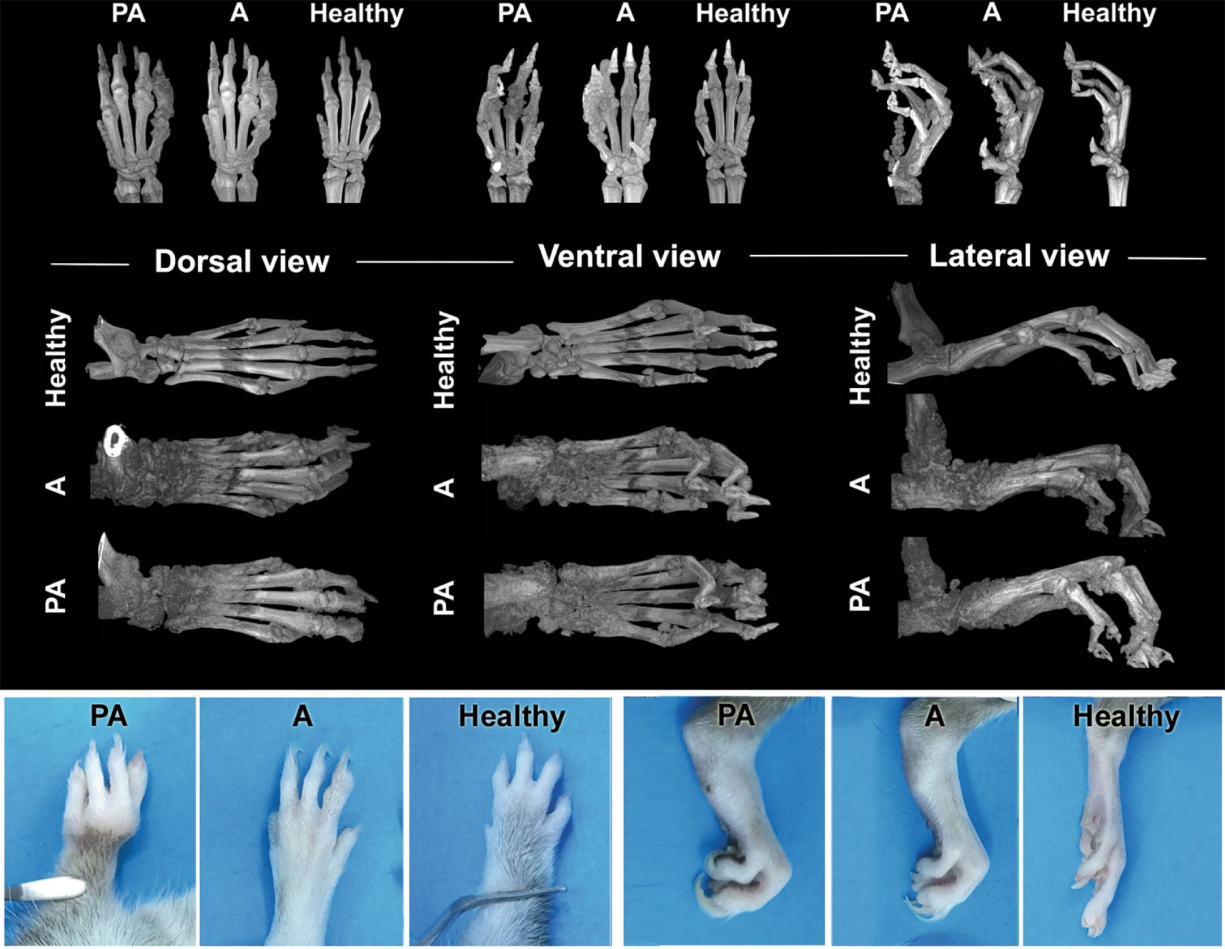
Micro-CT images and visual appearance of front and hind paws for assessment of arthritic changes. Micro-CT images of bone and tissue changes in front and hind paws of animals affected with experimental periodontitis and induced arthritis (PA), arthritis without periodontitis (A) and corresponding images of DA rats without induced arthritis or periodontitis (Healthy) at the time-point of 15 weeks; and visual appearance of front and hind paws at endpoint

To explore the impact of pre-existing periodontitis on the initiation, rate of progression, and severity of arthritis, the PIA model was adopted to quantify the contribution of periodontal infection in the disease process. Prior to immunization with pristane, animals with ligatures showed no signs of systemic illness and gained weight at the same rate as animals without experimental periodontitis (non-ligated) (Fig. 2a). All animals in the periodontitis group showed clinical signs of periodontal disease prior to injection of pristane (Fig. 1j). Mean arthritis score, based on evaluation of macroscopic inflammation (swelling and redness) in limbs, appeared around 2 weeks post pristane injection with a subsequently more severely developed arthritis leading at the end to a chronic relapsing disease course, as previously described [44]. The arthritis developed irrespectively of absence or presence of periodontitis, reaching maximum at 3–4 weeks post injection (Fig. 2b). For animals with periodontitis and arthritis (PA), the mean arthritis score (±SE) during experimental weeks 9–15 varied between 0.1 ± 0.1 and 23 ± 2.9, as compared to 0.1 ± 0.08 and 26 ± 1.9 for animals with arthritis only (A) (Fig. 2b). Micro-CT analysis of front and hind paws at the end of experiment confirmed our clinical findings showing that the pre-existence of periodontitis did not affect the severity of arthritis. Both PA and A groups showed markedly bone and cartilage changes compared to corresponding healthy animals as illustrated by micro-CT images in Fig. 3. Furthermore, the presence of periodontitis had no impact on mean weight change of pristane injected rats (Fig. 2a). Mean weight ± SE varied between 212 ± 9.7 and 251 ± 8.3 g in the PA group post pristane injection, as compared to 213 ± 6.2 and 257 ± 4.8 g in the A group. After 4 weeks post pristane injection (experiment week 12), the mean arthritis scores decreased (Fig. 2b) indicating a period of remission, at this period the animals also started to gain weight (Fig. 2a).

### Effect of pre-existing periodontitis on α-1-AGP, CRP and cytokine/chemokine response during pristane-induced arthritis

α-1-AGP is an acute phase protein that is effected by injury and/or infection [45]. Prior to pristane injection, mean plasma levels of α-1-AGP were comparable for animals affected with periodontitis (varying between a mean of 186 ± 43 and 334 ± 47 μg/ml) and periodontally healthy rats from the non-ligated arthritis group (varying between 214 ± 25 and 313 ± 28 μg/ml) (Fig. 2c). Levels of α-1-AGP increased rapidly in both groups after immunization and peaked at around 3 weeks after immunization, although no significant differences between groups were observed. However, a tendency (P = 0.07) towards higher plasma α-1-AGP levels in periodontitis-affected rats was seen at endpoint following pristane injection (mean: rats with periodontitis and induced arthritis, PA, 422 ± 65 to 1386 ± 250 μg/ml; rats with arthritis only, A, 358 ± 49 to 1010 ± 156 μg/ml) (Fig. 2c). Similar results were obtained when analyzing CRP levels showing increased CRP after the induction of PIA for both the PA (mean concentration 748 ± 45 μg/ml) and the A group (mean concentration 813 ± 72 μg/ml), with no significant differences between the groups. Moreover, no differences were observed between the PA and A group neither at baseline (510 ± 53 and 469 ± 110 μg/ml, respectively), nor at endpoint (525 ± 86 and 598 ± 53 μg/ml, respectively) (Fig. not shown).

Considering the importance of cytokine/chemokine interplay on development and progression of arthritis, we analyzed the levels of multiple cytokines in plasma. No significant difference in levels of: TNF-α, IL-1α, IL-1β, IL-2, IL-4, IL-5, IL-7, IL-10, IL-12, IL-13, IL-17, IL-18, EPO, GM-CSF, M-CSF, GRO/KC, MIP-3α, RANTES, VEGF and MCP-1 were observed (Table 1). Although the differences were not statistically significant, we observed that the mean levels of pro-inflammatory cytokines were consistently higher in the case of arthritic animals with accompanying periodontal disease (Table 1).

**Table 1:** Cytokine profile at endpoint in arthritis-affected DA rats with and without periodontitis

	PA (n = 9)	A (n = 12)	P value
TNF-α	110 (3–182)	75 (3–211)	NS
IL-1α	10,608 (2044–18,703)	10,299 (2191–29,782)	NS
IL-1β	20,842 (1170–45,925)	14,170 (1636–41,213)	NS
IL-2	1291 (532–2158)	1046 (282–2283)	NS
IL-4	723 (101–1223)	583 (124–1259)	NS
IL-5	566 (179–798)	469 (224–807)	NS
IL-7	3072 (418–4674)	2540 (526–7174)	NS
IL-10	899 (243–1274)	770 (205–1965)	NS
IL-12	985 (93–1939)	680 (120–1614)	NS
IL-13	243 (14–494)	170 (6–449)	NS
IL-17	214 (39–360)	173 (55–408)	NS
IL-18	2268 (1057–3765)	1449 (445–2673)	NS
EPO	780 (436–1079)	536 (145–1231)	NS
GM-CSF	259 (2–778)	127 (2–653)	NS
M-CSF	766 (578–1073)	753 (658–938)	NS
GRO/KC	214 (133–294)	150 (66–247)	NS
MIP-3α	227 (49–368)	175 (53–375)	NS
RANTES	1028 (402–1628)	735 (356–1280)	NS
VEGF	149 (23–286)	102 (20–246)	NS
MCP-1	4407 (1735–7249)	3634 (2499–4867)	NS

Mean (range) plasma concentrations (pg/ml) of cytokines (TNF-α, IL-1α, IL-1β, IL-2, IL-4, IL-5, IL-7, IL-10, IL-12, IL-13, IL-17, IL-18, EPO, GM-CSF, M-CSF, GRO/KC, MIP-3α, RANTES, VEGF and MCP-1) in arthritis rats with and without periodontitis, collected at endpoint

*DA* Dark Agouti, *PA* rats with experimental periodontitis and pristane-induced arthritis, *A* rats with pristane-induced arthritis without periodontitis, *NS* not significant

* P < 0.05 was considered statistically significant

### Effect of pre-existing periodontitis on the antibody response to RgpB and PPAD

**Fig. 4:**
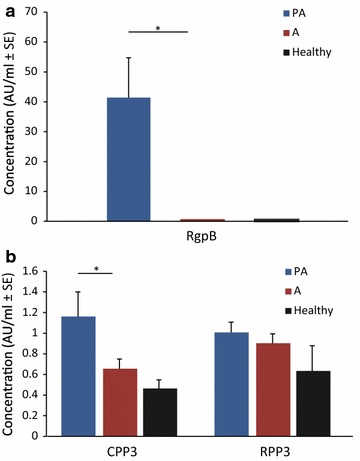
Plasma RgpB, CPP3 and RPP3 levels. Mean plasma concentrations (AU/ml ± SE) of antibodies against **a** RgpB, **b** CPP3 and RPP3 in arthritis rats with and without periodontitis and healthy control animals at endpoint of the experimental period of 15 weeks. *P < 0.05 was considered statistically significant. *PA* rats with experimental periodontitis and PIA (n = 9), *A* rats with PIA without periodontitis (n = 12), *Healthy* control rats without induced arthritis or periodontitis (n = 4), *RgpB* arginine gingipain B, *CPP3* citrullinated peptide derived from PPAD, *RPP3* arginine-containing (uncitrullinated) PPAD peptide

To confirm that challenging animals with *P. gingivalis* successfully elicited an immune response, we screened all plasma samples for the presence of antibodies against RgpB. Not surprisingly, considering our clinical findings, the majority (8 of 9) of the animals in the periodontitis and arthritis group (PA) were positive for antibodies against RgpB, whereas no anti-RgpB antibodies were detected in the arthritis group without periodontitis (A) or in the healthy control group (Fig. 4a).

In order to investigate the capacity of *P. gingivalis* to generate antibodies against citrullinated α-enolase and PPAD in the periodontitis ligature model, an ELISA-based approach was used. Antigens included a synthetic citrullinated peptide derived from the C-terminal region of PPAD (CPP3) [46, 47] and the immunodominant CEP-1 peptide derived from citrullinated human α-enolase [48]. Animals in the PA group had significantly (P < 0.05) higher levels of antibodies to CPP3 as compared to the arthritis group, while there were no differences in antibody response to the corresponding arginine-containing (i.e. non-citrullinated) control peptide RPP3 between these groups (Fig. 4b). Moreover, the levels of CPP3 and RPP3 did not significantly differ between the arthritis-only animals (A) and the healthy control rats (Fig. 4a, b). None of the animals were positive for antibodies to CEP-1 or the non-citrullinated control REP-1 peptide (data not shown).

## Discussion

There is growing evidence of a link between RA and periodontitis reflected in shared chronic nature of the inflammatory responses, as well as a number of environmental factors, including smoking and the periodontal pathogen *P. gingivalis* [49]. In this study we demonstrate, for the first time, the presence of antibodies to citrullinated peptide derived from PPAD (CPP3) in rats with ligature and periodontal pathogen-induced periodontitis and experimental arthritis compared to periodontitis free animals with arthritis.

To evaluate the impact of pre-existing periodontitis on the development of PIA, a well-established ligature model of experimental periodontitis combined with swabs containing the oral pathogens *P. gingivalis* and *F. nucleatum* was used [50, 51]. This combination of bacteria was used because the periodontitis-associated *P. gingivalis* has been implicated in the development of arthritis and *F. nucleatum* is known to promote the colonization of *P. gingivalis* adjacent to the affected teeth [11, 17]. In addition, polymicrobial infection with these two bacteria has been shown to induce a stronger inflammatory response compared with infection with either bacterium alone [52]. In the current study, severe localized periodontitis, as assessed by tooth mobility, manifested prior to PIA-induction in all experimental animals and the loss of alveolar bone was confirmed by intraoral radiographs. The assessment of clinical parameters such as tooth mobility to study the manifestation of periodontal disease has previously been described in animals [38] and is frequently used in humans [53]. The clinical manifestations of arthritis in our study were observed after 2–3 weeks post pristane injection in all animals, followed by a decrease in arthritis disease activity, as previously described [44]. The pre-existence of periodontitis did not affect the development or disease severity of pristane-induced arthritis. In line with our results, Trombone et al. reported no additional effect of periodontitis co-induction on the severity of pristane-induced arthritis in mice [54]. Conversely, subcutaneous infection with *P. gingivalis* has been reported to exacerbate both collage-induced arthritis (CIA) in mice and adjuvant arthritis in female DA rats [31, 55]. The conflicting results in arthritis severity by periodontitis co-induction may depend on different experimental models of arthritis and periodontitis. Most animal studies that report a more severe arthritis by periodontitis co-induction have used CIA or collagen antibody-induced arthritis (CAIA) [55–58]. Furthermore, different strains of *P. gingivalis* could also affect the results, as not all *P. gingivalis* strains are equally effective in promoting bone loss [59] and could also differ in their ability to exacerbate arthritis. In the present study the well-established animal model of arthritis, the PIA model in the rat, was chosen based on its close mimicry to human RA [34]. PIA triggers a chronic relapsing arthritis that fulfills many of the RA criteria such as symmetrical involvement of peripheral joints, presence of rheumatoid factor, cartilage and bone destruction, as well as a chronic course of the disease [60]. The arthritis is associated with MHC class II genes [61] and mediated by MHC class II restricted T cells [62, 63] as well as with a strong innate immune response [64]. Because the disease course in RA can exhibit two different patterns; a chronic-persistent [65, 66] or a relapsing-remitting [66, 67], the experimental period in this study was set to 15 weeks in order to monitor the whole course of the disease. To our knowledge, this study with a duration of 15 weeks is the longest animal study investigating the effects of experimental periodontitis on the disease course of arthritis, including both active and chronic arthritis.

The oral pathogen *P. gingivalis*, strongly associated with periodontitis, has been suggested to be involved in the pathogenesis of RA since the presence of *P. gingivalis* DNA has previously been reported in the synovial tissue of RA patients [43], and since patients with RA have been shown to have higher anti-*P. gingivalis* and anti-RgpB antibody levels, compared to controls [26, 30]. In the current study, in line with previously reported data [68] *P. gingivalis* DNA was detected in the oral cavity of the periodontitis-affected animals, although the presence of *P. gingivalis* in metacarpophalangeal joint tissue of periodontitis-affected animals could not be detected. To our knowledge, *P. gingivali*s DNA in synovial tissue in experimental animal models has not yet been reported. In addition, we were not able to detect *P. gingivalis* in cultures of oral swabs and ligatures from infected animals at endpoint, which may be due to the strict anaerobic nature of this pathogen [69], being more sensitive to normal levels of oxygen, *P. gingivalis* may be less likely to survive the long transportation period in our study (up to 8–9 h due to practical reasons) for re-cultivation purposes. This suggestion is also in line with previous findings reporting that only 50% of *P. gingivalis* are viable after 2 h of incubation (5% CO_2_ at 37 °C) and none may survive for 8 h [70]. The reigning hypothesis linking periodontitis to RA suggests that *P. gingivalis´* ability to secrete the PAD enzyme, PPAD, may contribute to the initiation of RA by triggering citrullination of proteins in the gingival tissue and break immune tolerance, with the subsequent production of ACPA. Epitope spreading to other host-citrullinated proteins in the joint could then perpetuate an inflammatory process and cause RA [17, 49]. In support of this hypothesis, the results from histological studies have demonstrated that oral inoculation with *P. gingivalis* results in significantly more bone and cartilage destruction in joints, and higher number of osteoclasts and inflammatory cells in the periodontium of arthritic mice, as compared to animals without *P. gingivalis* infection or *P. gingivalis* strain lacking the PAD enzyme [56, 58]. Considering the central role of citrullinated proteins and ACPAs in the etiopathogenesis of RA, and the findings suggesting that ACPAs may also have a significant impact on the development and progression of arthritis in experimental animal models [26, 71], we have—in the current study—analyzed the presence of antibodies against two citrullinated peptides; CPP3 derived from PPAD and CEP-1 derived from α-enolase, as well as antibodies against RgpB, one of *P. gingivalis* most potent virulence factors, crucial for PPAD citrullination [25, 72, 73]. Our results showed that 89% of the periodontitis-affected animals were positive for antibodies against RgpB. Moreover, animals with induced periodontitis and arthritis had significantly higher levels of autoantibodies against CPP3, as compared to animals with arthritis only or healthy controls. The presence of citrullinated proteins in the inflamed synovium of arthritis-affected animals has previously been reported in different animal models [74–76], and one study reported a non-significant increase in serum ACPA levels in co-morbid mice challenged with both periodontitis (induced by oral inoculation with wild-type *P. gingivalis*) and arthritis, compared to corresponding animals infected with PAD-deficient *P. gingivalis* [58]. A significant increase in ACPA levels has also been reported in arthritic mice, after a subcutaneous *P. gingivalis* infection, compared to mice infected with a PPAD-knockout strain [55]. However, one should keep in mind that animals may express autoantibodies also against the corresponding non-citrullinated peptides, making the antibody response not necessarily citrulline-specific but rather directed against other parts of the peptide, highlighting the importance of uncitrullinated negative control antigens when working with animal models [77]. Importantly, in our study, significantly higher antibody levels in co-morbid rats were only detected against CPP3, not against RPP3, as compared to the arthritis group. However, we did not detect any antibodies against CEP-1 or the uncitrullinated control peptide REP-1 (data not shown).

The acute phase proteins α-1-AGP and CRP are normally elevated in plasma as a result of injury, infection or inflammation [45, 78]. In patients with RA, α-1-AGP positively correlate with erythrocyte sedimentation rate (ESR), disease activity and the degree of disability, and has therefore been suggested to be a useful biochemical marker for evaluation of RA disease activity [79]. In rats with pristane-induced arthritis, the α-1-AGP levels also correlate well with arthritis development [64]. In the present study, animals with co-morbid periodontitis and arthritis exhibited non-significantly higher plasma α-1-AGP at endpoint compared to the arthritis group without periodontitis, suggesting a more acute systemic inflammation. Concentrations of α-1-AGP followed the same pattern in all animals, remaining stable prior to pristane injection, irrespective of periodontal status, and increased several-fold after arthritis-induction. The levels of α-1-AGP were also reflected by the clinical manifestations of arthritis progression. The rapid initial increase in plasma α-1-AGP levels with a peak after 3 weeks of post arthritis-induction indicated a period of acute inflammation followed by a decrease during the time of remission of the first acute phase, irrespective of periodontal disease. Additionally, the levels of CRP, previously reported to be associated with periodontitis and with anti-*P. gingivalis* antibody levels in patients with RA [2, 21, 80], were also significantly increased both in PA and A group post PIA-induction with no significant differences between the two groups. Increased levels of α-1-AGP and CRP during arthritis in rats have previously been reported [64, 81], however, to our knowledge this is the first study reporting the levels of α-1-AGP in co-morbid rats with also experimental periodontitis.

Cytokines and chemokines are important key molecules and signal mediators in the pathogenesis of RA and periodontitis [19, 82]. Their disease-relevant functions include activation of immune cells, fibroblasts, osteoclasts and release of proteolytic enzymes that collectively contribute to bone and tissue destruction in both RA and periodontitis [19, 82]. Several of the cytokines investigated in the present study have been demonstrated to serve either as pro-inflammatory (IL-1, IL-12, IL-17, IL-18, TNF-α) or anti-inflammatory (IL-4, IL-10) mediators of the inflammatory process, or chemokines involved in inducing chemotaxis and attracting immune cells to the inflammation site (MCP-1, RANTES) [82]. In the plasma samples of DA rats, we could not show significant differences in the cytokine profiles following periodontitis/PIA co-induction. Notably, mean concentrations of the investigated cytokines from the animals with coexisting periodontitis and arthritis were higher than in periodontitis free animals, suggesting increased activation of immune system due to additional inflammatory stimuli. In mice, *P. gingivalis* induced periodontitis and PIA co-induction has been reported to increase the levels of IL-1ß and IL-17 in periodontal tissue [54]. It is tempting to speculate that observed increase in the immune system activity might in the long run lead to breach of self-immunity and development of autoimmune disease.

There are, however, potential limitations with the current study we need to take into account. First, the experimental group with periodontitis alone was not included in the experiments since the focus of this study was to investigate the effect of pre-existing periodontitis on the development of arthritis rather than the effect of arthritis on periodontitis. In this context, previous studies have showed that experimentally induced arthritis also increase periodontal bone loss in rats and mice [32, 56, 58]. Secondly, no definite interpretations can be drawn about the specific effect of *P. gingivalis* on the development of arthritis, since the experimental periodontitis was induced by ligatures in combination with two periodontal pathogens to obtain a severe periodontitis and promote the *P. gingivalis* infection (by coaggregation with *F. nucleatum*) [11]. Finally, the rapid induction and severity of the PIA model may be too strong, potentially masking the effects of periodontitis on arthritis development. However, the CIA model, also used to investigate the periodontitis-arthritis association in mice, is in comparison similar or even more severe than the PIA in rats [83]. In this study, a standardized protocol for PIA-induction was chosen, previously used by our group and others, to reduce the risk of variability and increase the possibility of detecting significant effects between the study groups [35, 54].

## Conclusions

In conclusion, these results suggest that animals with arthritis and pre-existing periodontitis, induced by ligatures in combination with *P. gingivalis* and *F. nucleatum* infection, generate higher systemic immune response and antibody titers against RgpB and PPAD citrullinated peptides, without affecting arthritis disease severity. To our knowledge, this is the first animal study reporting significantly increased antibody response to PPAD citrullinated proteins, following a local oral infection with *P. gingivalis*. The infection by *P. gingivalis* could potentially be the underlying mechanism connecting periodontitis to RA, by giving rise to a systemic immune response against citrullinated proteins, with generation of RA specific antibodies/ACPAs.

## Additional file

**Additional file 1: Figure** **S1.** Detection of bacterial DNA. **a** Total load of non-species-specific bacterial gDNA and **b** specific *P. gingivalis* DNA (relative quantification) from ligatures of rats with pre-existing periodontitis, collected at endpoint. DNA from *P. gingivalis* CCUG 14449 strain; *Positive Ctrl* positive control, *NTC* non-templated control (PCR-graded water).

## Abbreviations

RA: rheumatoid arthritis
DA: Dark Agouti
PIA: pristane-induced arthritis
PA: experimental periodontitis animals with induced arthritis
A: arthritis induced animals without periodontitis
*P. gingivalis*: *Porphyromonas gingivalis*
*F. nucleatum*: *Fusobacterium nucleatum*
FAB: fastidious anaerobe broth
α-1-AGP: α-1-acid glycoprotein
CRP: C-reactive protein
PPAD: *P. gingivalis* peptidylarginine deiminase
ACPA: anti-citrullinated protein antibody
RgpB: arginine gingipain B
CPP3: citrullinated peptide derived from PPAD
RPP3: arginine-containing (uncitrullinated) PPAD peptide
CEP-1: citrullinated α-enolase peptide 1
REP-1: arginine-containing (uncitrullinated) α-enolase peptide 1
gDNA: genomic DNA
NTC: non-templated control
Micro-CT: micro-computed tomography
NS: not significant
