# Supplementary figures and images for: Effects by periodontitis on pristane-induced arthritis in rats
Source: J Transl Med. 2016 Nov 3;14:311. doi: 10.1186/s12967-016-1067-6 (PMC5094068; doi:10.1186/s12967-016-1067-6)

**A**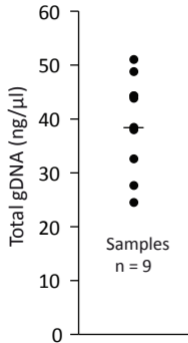**B**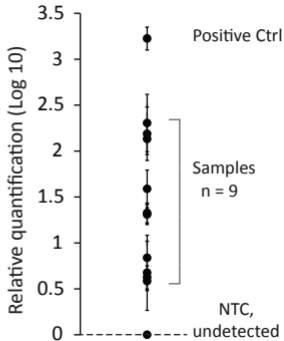

Supplement: Supplementary file 1 — Additional file 1: Figure S1. Detection of bacterial DNA. a Total load of non-species-specific bacterial gDNA and b specific P. gingivalis DNA (relative quantification) from ligatures of rats with pre-existing periodontitis, collected at endpoint. DNA from P. gingivalis CCUG 14449 strain; Positive Ctrl positive control, NTC non-templated control (PCR-graded water). [file 12967_2016_1067_MOESM1_ESM.pdf]
